# Effect of high intakes of protein-only and carbohydrate-only on plasma metabolites and hormones, in addition to nitrogen excretion

**DOI:** 10.3389/fendo.2025.1618142

**Published:** 2025-08-11

**Authors:** Matthieu Clauss, Claire Puissant, Nasser Ezzatkhah Bastani, Lotte L. K. Nielsen, Bjørn Steen Skålhegg, Per Bendix Jeppesen, Jørgen Jensen

**Affiliations:** ^1^ Department of Physical Performance, Norwegian School of Sport Sciences, Oslo, Norway; ^2^ Department of Nutrition, Division for Molecular Nutrition, University of Oslo, Oslo, Norway; ^3^ Department of Clinical Medicine, Aarhus University Hospital, Aarhus, Denmark

**Keywords:** insulin, glucagon, incretin, GLP-1, GIP, time course, urinary nitrogen excretion, protein metabolism

## Abstract

**Background:**

Hormonal and metabolic responses to high protein intake are not well understood. The aims of this study were to compare the metabolic and hormonal responses to isocaloric intakes of whey protein alone versus carbohydrate alone. Additionally, we measured urinary nitrogen excretion as a marker of protein degradation.

**Methods:**

Fourteen young, healthy, moderate-to-well-trained participants (VO_2max_ 50.6 ± 2.9 mL·kg^-1^·min^-1^; mean ± SEM) reported in the morning after an overnight fast. In a double-blinded, randomized, balanced cross-over design, participants consumed isoenergetic test drinks containing either 1.2 g·kg^-1^ of whey protein alone (PRO) or carbohydrate alone (CHO) on separate days. They recorded their dietary intake the day before and during the intervention to repeat them across the second trial day. Blood samples were collected at regular intervals after drink ingestion. Urine was collected throughout the testing period in six consecutive batches.

**Results:**

After CHO intake, plasma glucose levels increased, and certain plasma amino acid concentrations decreased. Following PRO intake, plasma glucose decreased, and plasma amino acids increased. Insulin concentrations increased following ingestion of both CHO and PRO (time effect, p<0.001), with a greater increase in CHO (drink effect, p<0.001). Plasma GLP-1 and GIP concentrations increased in both conditions (time effect, p<0.001). Plasma GLP-1 increased more in PRO than in CHO (drink effect, p<0.001), whereas plasma GIP increased more in CHO than in PRO (drink effect, p<0.001). Urinary nitrogen excretion over the 24 hours following drink ingestion was significantly higher in PRO (p<0.001), particularly between 2 to 8 hours after intake (p<0.001).

**Conclusions:**

CHO increased plasma insulin more than PRO. The PRO induced insulin response was independent of glucose and mediated by the increase in plasma amino acids and GLP-1. Interestingly, the GLP-1 response was larger following PRO and remained elevated after 240 minutes, whereas the GIP response was larger following CHO. Additionally, protein-only ingestion increased urinary nitrogen excretion, mainly between 2 to 8 hours after intake, with elevated excretion persisting up to 24 hours.

## 1 Introduction

Protein is an essential nutrient with a daily requirement of 0.83 g kg^-1^ d^-1^ for adults (1). However, protein metabolism is adaptable and endurance athletes may require higher amounts, ranging from 1.6 to 1.8 g kg^-1^ d^-1^ (2–5). Amino acids (AAs) are necessary for protein synthesis and for the production of various metabolites (1, 6). In addition, they can be metabolized for energy production. Typically, the metabolic and hormonal responses to protein intake have been studied in combination with carbohydrates and/or fats (7–10). In contrast, the metabolic responses to glucose intake have been extensively studied through oral glucose tolerance tests and glucose infusion (11–13).

Glucose intake stimulates insulin secretion, by increasing plasma glucose concentrations (14, 15) and by promoting the secretion of glucagon-like peptide-1 (GLP-1) and glucose-dependent insulinotropic polypeptide (GIP) from the gut (16–18). The insulin response is two to three times higher when the same quantity of glucose is ingested orally compared to direct infusion into the bloodstream (19, 20). GLP-1 and GIP contribute to 50–70% of postprandial insulin secretion following carbohydrate consumption (21, 22).

Protein intake leads to a temporary increase in plasma AA concentrations (23, 24). *In vitro* studies have shown that AAs, particular arginine, stimulate insulin secretion from beta-cells (25). Co-ingestion of protein with carbohydrate has been shown to further elevate plasma insulin concentrations compared to carbohydrate alone (24, 26, 27). Similarly, studies investigating protein-only ingestion have shown that insulin concentrations increase proportionally to the amount of protein consumed (16, 28–30). Protein ingestion also stimulates glucagon secretion, which contributes to counter-regulation and helps maintain plasma glucose levels (31). The effect of protein on GLP-1 and GIP responses in humans has only been studied with small protein quantities (16, 30) or co-ingested with other nutrients (7–10). These studies show minimal differences in insulin response and making their contribution difficult to quantify. Furthermore, comparisons of protein-only and carbohydrate-only intakes are scarce and have not always been isocaloric (16). Therefore, the impact of protein consumption on insulin response warrants further investigation, particularly with larger protein quantities.

Proteins are not stored in the body, and the capacity to store AAs is limited (32, 33). When AAs ingested are not used for protein synthesis or synthesis of nitrogenous metabolites, they are metabolized in the liver, where their nitrogen is incorporated into urea, whereas their carbon skeletons are metabolized or transformed into glucose or fat (34). As a result, amino metabolism strongly depends on protein intake (33). Since nitrogen from AAs is mainly excreted in urine, urinary nitrogen excretion serves as an indicator of this metabolic process. Traditionally, urinary nitrogen excretion has been measured over a 24-hour period for medical purposes (35). Therefore, the time resolution and interpretation of nitrogen excretion of shorter duration is uncertain.

A major problem in exercise studies is that the time resolution of nitrogen excretion is not well established (36). Previous research has shown that nitrogen excretion increases rapidly after protein intake following exercise (24, 37, 38). For example, urinary nitrogen excretion was higher in the hours after consuming a carbohydrate-protein drink during recovery from exhaustive exercise compared to a carbohydrate-only drink (24). However, this increase was less than the quantity of protein ingested. Understanding the time resolution of nitrogen excretion can help interpret this difference.

The aim of the present study was to compare the plasma responses of hormones and metabolites, as well as urinary nitrogen excretion, following isocaloric intake of either whey protein or carbohydrates. Specifically, the study aimed to investigate the responses of plasma glucose, AAs, insulin, glucagon, and incretins after the intake of either 1.2 g·kg^-^¹ of protein alone or carbohydrate alone. Additionally, we examined the time course of urinary nitrogen excretion when a large quantity of AAs is readily available.

## 2 Materials and methods

### 2.1 Participants

Fourteen young healthy participants (6 women and 8 men, age range 20 to 32 years) were recruited for the study. Their characteristics are described in **Table 1**. Participants were informed about the study before providing their written informed consent. In addition, all participants completed a health questionnaire to rule out potential risk factors. The Norwegian School of Sport Sciences Ethic Committee (Application 197-020921) and the Norwegian Centre for Research Data (Reference number 435376) approved the study, which conformed to the standards set by the latest revision of the Declaration of Helsinki.

**Table 1:** Participants characteristics.

Parameter	Women and men (n=14)	Women (n=6)	Men (n=8)
Age (years)	26.6 ± 0.9	26.4 ± 1.7	26.8 ± 1.0
Bodyweight (kg)	75.2 ± 3.2	69.4 ± 6.3	79.5 ± 2.3
Height (m)	1.77 ± 0.02	1.70 ± 0.01	1.81 ± 0.02 *
BMI (kg·m^-2^)	24.0 ± 0.9	23.8 ± 1.9	24.2 ± 0.7
Lean mass (kg)	58.8 ± 2	52.5 ± 2.9	63.5 ± 0.8 *
Fat mass (kg)	16.3 ± 2	17.8 ± 4.0	15.2 ± 1.9
Fat mass (%)	21.7 ± 1.8	25.0 ± 3.3	19.2 ± 1.8
VO_2max_ (L·min^-1^)	3.8 ± 0.2	3.3 ± 0.2	4.1 ± 0.3 *
VO_2max_ (mL·kg^-1^·min^-1^)	50.6 ± 2.9	49.1 ± 4.3	51.7 ± 4.0
HR_max_ (beat·min^-1^)	193.6 ± 2.5	199.0 ± 3.4	189.5 ± 3.0

Data are given as average ± SEM. BMI body mass index; VO_2max_ maximal oxygen uptake; HR_max_ maximal heart rate. *p < 0.05, parameters with significant differences between women and men.

### 2.2 Study design

**Figure 1:**
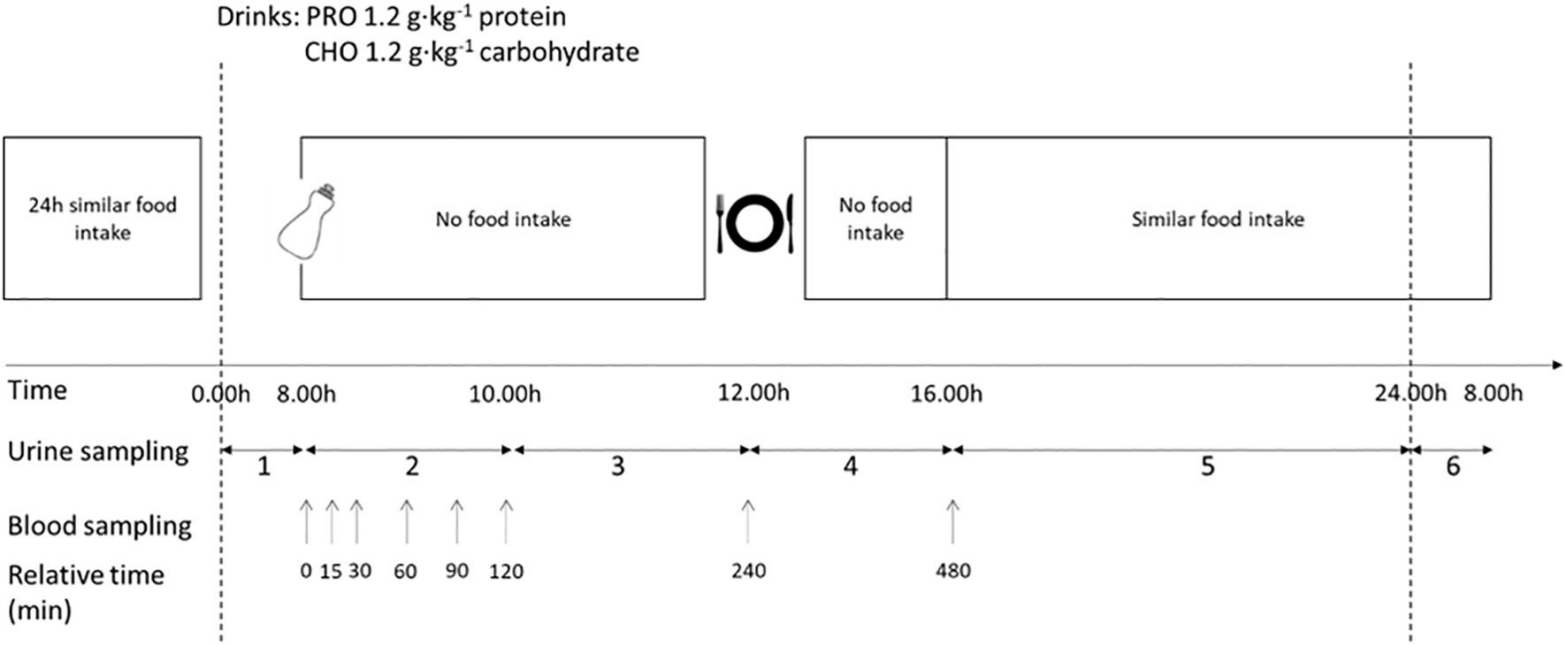
Schematic overview of the design of the intervention. The protocol was completed in a randomized, balanced crossover experimental design. The study consisted of two testing days with a carbohydrate-only drink (CHO) or protein-only drink (PRO). No physical activity was performed, and the diet was repeated during the two trial days. The urine collection periods are labelled in the figure.

This study was a randomized, balanced, and double-blinded cross-over experiment (**Figure 1**). The study consisted of two interventions in which the effect of ingesting protein-only or carbohydrate-only on hormone responses and urinary nitrogen excretion was tested. The participants performed some initial tests to determine physiological parameters.

#### 2.2.1 Preliminary tests

##### 2.2.1.1 Maximal oxygen uptake

On the first day of the study, the participants’ maximal oxygen uptake (VO_2max_) was assessed. The VO_2max_ test was conducted on a cycle ergometer (Lode Excalibur, Lode, Groningen; The Netherlands) in a controlled, distraction-free environment with similar ambient conditions (18-19°C). Participants were required to maintain a pedaling frequency of ~75 rpm throughout the test. Participants performed an incremental warm-up before VO_2max_ was measured. The VO_2max_ test started at the second last step of the incremental warm-up, with intensity increasing by 25 W steps every 60 seconds until participants reached voluntary exhaustion. VO_2_ was measured continuously (Oxycon Pro; Jager Instruments, Hoechberg, Germany). VO_2max_ was defined as the mean of the two highest consecutive 30-s epochs. Heart rate (Polar RS800CX, Kempele, Finland) was measured continuously throughout the VO_2max_ test. The maximal heart rate (HR_max_) was the highest heart rate reached during this test.

##### 2.2.1.2 Dual-energy X-ray absorptiometry (DEXA) body scan

Body composition was measured using a whole body Dual-Energy-X-ray Absorptiometry (Scanex Medical Systems AS, GE Lunar iDXA, GE Healthcare, program ENCORE 18). Fat, lean, bone (bone mineral content), and total body mass were determined. The subjects were instructed to arrive at the laboratory in a fasted state and to abstain from exercise for 12 hours prior to each laboratory visit.

#### 2.2.2 Intervention days

Before the intervention days, participants were instructed to refrain from exercise during the 24 hours preceding each test. They recorded their dietary intake during the 24 hours prior to the first test and replicated the same intake before the second intervention day.

Participants reported to the laboratory early in the morning (07.45 a.m.) following an overnight fast that began after dinner the previous evening. A venflon (BD VeneflonTM Pro, Helsingborg, Sweden) was inserted into the medial cubital vein, and a fasting blood sample was collected.

##### 2.2.2.1 Treatment drink

The participants were given either a protein-only or a carbohydrate-only drink in a randomized order. The two drinks were isoenergetic. The PRO drink was prepared by dissolving whey isolate (100% Whey Isolate raspberry taste, Scitec Nutrition, Australia) in water to achieve a 20% drink (in weight), providing 1.2 g·kg^-1^ protein. The bodyweight measured at the start of the preliminary test was used to determine the appropriate amount. The nutritional values for the whey isolate used were as follows (per 100 g of powder): energy 348 kcal, protein 84 g, carbohydrate 2.4 g, lipid 0.4 g. The amino acid profile provided by the manufacturer was as follows (per 100 g of powder): histidine 1.4 g, isoleucine 4.4 g, leucine 8.1 g, lysine 7.0 g, methionine 1.8 g, phenylalanine 2.4 g, threonine 4.9 g, tryptophan 1.3 g, valine 4.2 g, arginine 3.0 g, cysteine 2.4 g, glutamine and glutamic acid 18.7 g, proline 4.2 g, tyrosine 2.3 g, alanine 3.7 g, asparagine and aspartic acid 7.9 g, glycine 1.4 g, serine 3.6 g. The CHO drink consisted of 0.6 g·kg^-1^ glucose (GPR RECTAPUR^®^, VWR Chemicals, Radnor, Pennsylvania, USA) and 0.6 g·kg^-1^ maltodextrin (Dextri-maltose^®^, MP Biomedicals, Santa Ana, California, USA) in flavored water [100 g L^-1^ of Fun Light Raspberry (Stabburet, Norway)] to achieve a 20% drink (in weight). All drinks contained identical quantities of electrolytes (0.7 g L^-1^ NaCl) and were indistinguishable in taste.

##### 2.2.2.2 Blood analyses

Blood samples were collected prior to consumption of the drink and then at 15, 30, 60, 90, 120, and 240 minutes following ingestion. After each blood test, the venflon was rinsed with sterile saline solution (NaCl 0.9%, B. Braun Melsungen AG, Germany). Samples were collected in 6 ml K2E K2EDTA tubes (Vacuette tubes, Greiner Bio-One, Kremsünster, Austria). Samples were centrifuged (Eppendorf centrifuge 5702 R) at 2500 g and 4°C for 10 minutes immediately after blood collection. In case immediate centrifugation was not possible, the tubes were stored on ice until centrifugation could be performed. The samples were then pipetted into 1.5 ml Eppendorf tubes (Protein LoBind tube, Eppendorf AG, Hamburg, Germany) and stored at -80°C until analysis.

Concentrations of lactate and glucose were measured in blood drawn from the venflon. A drop of blood from the syringe was placed onto a sterile plastic pad to fill a 55 μL capillary tube (Radiometer, Copenhagen, Denmark). The sample was analyzed using Biosen C-Line Lactate analyzer (EKF Diagnostics, UK).

##### 2.2.2.3 Food intake

No food was consumed during the 4 hours following ingestion of the drink. Following the blood sample collection at 240 minutes, a standardized low-protein meal was provided (**Table 2**). To prevent significant deviations in protein intake from participants’ habitual diets, they were allowed to consume food of their choice in the afternoon after 16.00. However, they were instructed to replicate the same food intake on the second intervention day. Total energy (kcal), carbohydrate, protein, and fat intakes were calculated using the Kostholdsplanleggeren program (Norwegian Directorate of Health, Norwegian Food Safety Authority, Norway).

**Table 2:** Energy intake and content of macronutrients for the standardized meals during the study protocol.

Time period	Energy intake (kcal)	Carbohydrate intake (g kg^-1^)	Protein intake (g kg^-1^)	Fat intake (g kg^-1^)
Day before	2683.7 ± 214.1	3.76 ± 0.19	1.59 ± 0.19	1.47 ± 0.21
Intervention day				
Drink ingestion: 8.00 (CHO/PRO)	362.9 ± 3.0	1.20 ± 0.01 / 0 ± 0	0 ± 0 / 1.20 ± 0.01	0 ± 0
Period 4: 12.00 to 16.00	801.5 ± 73.3	1.82 ± 0.16	0.25 ± 0.03	0.25 ± 0.04
Period 5: 16.00 to 24.00	1505.1 ± 269.2	2.17 ± 0.43	0.93 ± 0.12	0.77 ± 0.13
Period 6: 00.00 to 08.00	107.9 ± 75.9	0.19 ± 0.14	0.06 ± 0.04	0.05 ± 0.04
Total macronutrient (CHO/PRO)	2775.3 ± 252.9	5.37 ± 0.39 / 4.17 ± 0.39	1.24 ± 0.12 / 2.44 ± 0.12	1.07 ± 0.14

The total macronutrient amount for CHO and PRO is calculated from all food intake during the urine collection periods (Periods 1-6) and test drink consumption.

Participants were asked not to exercise during the intervention day.

The last blood sample was collected 8 hours after the ingestion of the drink.

Additionally, participants were asked to record any food intake the following morning before the end of the urine collection period at 8.00 a.m.

##### 2.2.2.4 Urine collection

During the intervention days, urine samples were collected in plastic containers in six consecutive batches: 1) from midnight until ingestion of the drink (duration ~8h, from 00.00 to 08.00), 2) the first 2 hours after the ingestion of the drink (2h, from 08.00 to 10.00), 3) the next 2 hours (2h, from 10.00 to 12.00), 4) the 4 hours after lunch (4h, from 12.00 to 16.00), 5) from the time the intervention session concluded until midnight (~8h, from 16.00 to 24.00), and 6) from midnight to 8:00 the next day (8h). Volume was measured for each period, and a 15-ml sample was frozen at −20°C for analysis.

##### 2.2.2.5 Calculation of nitrogen balance

The nitrogen concentration in the urine was analyzed with the Kjeldahl method (39). Nitrogen intake was calculated assuming a nitrogen-protein constant of 6.25 (40). When estimating nitrogen excretion in all compartments, urinary nitrogen excretion was considered to represent 78% of total nitrogen excretion (4, 37). The nitrogen balance was calculated based on protein intake and nitrogen excretion in all compartments extrapolated from urinary nitrogen excretion. These calculations were carried out over 24 hours for the protocol period after consuming the drinks.

##### 2.2.2.6 Plasma amino acid concentrations

Plasma AAs were quantified using liquid chromatography–tandem mass spectrometry (LC-MS/MS). Briefly, isotopically-labelled internal standards were added to plasma, followed by reduction of disulfides using dithioerythritol and then protein precipitation using 5-sulfosalicyclic acid. The extracts were diluted with an aqueous solution of formic acid [0.5%] and heptafluorobutyric acid (HFBA) [0.3%] prior to analysis. LC-MS/MS was carried out using a Shimadzu LC-20ADXR Prominence LC system (Kyoto, Japan) coupled to a Sciex QTRAP 6500^+^ mass spectrometer with a Turbo V ion source and TurboIonspray probe (Framingham, MA, USA). Chromatographic separation was achieved on a Phenomenex Kinetex Core Shell C18 (100 x 4.6 mm, 2.6 μm) LC column (Torrance, CA, USA) with an aqueous solution of formic acid [0.5%] and HFBA acid [0.3%] and acetonitrile gradient mobile phase. Positive mode multiple reaction monitoring was used for detection. Linear calibration curves of the peak area ratios of analyte and internal standard were used for quantification. Coefficient of variation for the analytes were 3.4-6.7%.

##### 2.2.2.7 Plasma hormones and incretins concentrations

Plasma insulin concentration was measured with an enzyme-linked immunosorbent assay human insulin kit, K6219 (Dako, Glostrup, Denmark). Plasma glucagon concentration was quantified using an ELISA kit (Mercodia Glucagon ELISA kit 10-1271-01, Mercodia, Uppsala, Sweden). Plasma GLP-1 concentration was quantified using an ELISA kit (Mercodia Total GLP-1 ELISA kit 10-1278-01, Mercodia, Uppsala, Sweden). Plasma GIP concentration was quantified using an ELISA kit (Mercodia Total GIP ELISA kit 10-1258-01, Mercodia, Uppsala, Sweden).

##### 2.2.2.8 Plasma free fatty acid concentration

Plasma free fatty acid (FFA) concentration was measured by an enzymatic colorimetric assay for the quantitative determination of FFAs (Wako Chemicals GmbH, Neuss, Germany) using the autoanalyzer Cobas C-111 (Roche Diagnostics, Rotkreuz, Switzerland) (41).

### 2.3 Statistics

The study results were analyzed using Prism 9 (GraphPad Software, LLC). Two-way ANOVA (time x drink) with repeated measurements was conducted to analyze the data. Statistical tests were performed to statistically assess the equality of variance assumption before conducting ANOVA. All performed ANOVA passed the test. After identifying a significant effect, *post hoc* analyses were performed with Bonferroni corrections. In instances of missing data, mixed-effect analyses were applied. Cohen’s *d* effect size was calculated by dividing the difference between the two means by the pooled standard deviation (42). Paired t-tests were used to compare the effect of the drink on urinary nitrogen excretion and nitrogen balance. Principal component analyses were performed with the function PCA and correlation analyses were performed with the function cor.test from the ‘corrr’ library in R (Version 4.3.2, Vienna, Austria, 2023). The significance level was set to p ≤ 0.05. Statistical trends are defined as p-values between 0.05 and 0.10. Data are presented as mean ± standard error of the mean (SEM) in text, figures, and tables.

## 3 Results

### 3.1 Plasma glucose concentration

**Figure 2:**
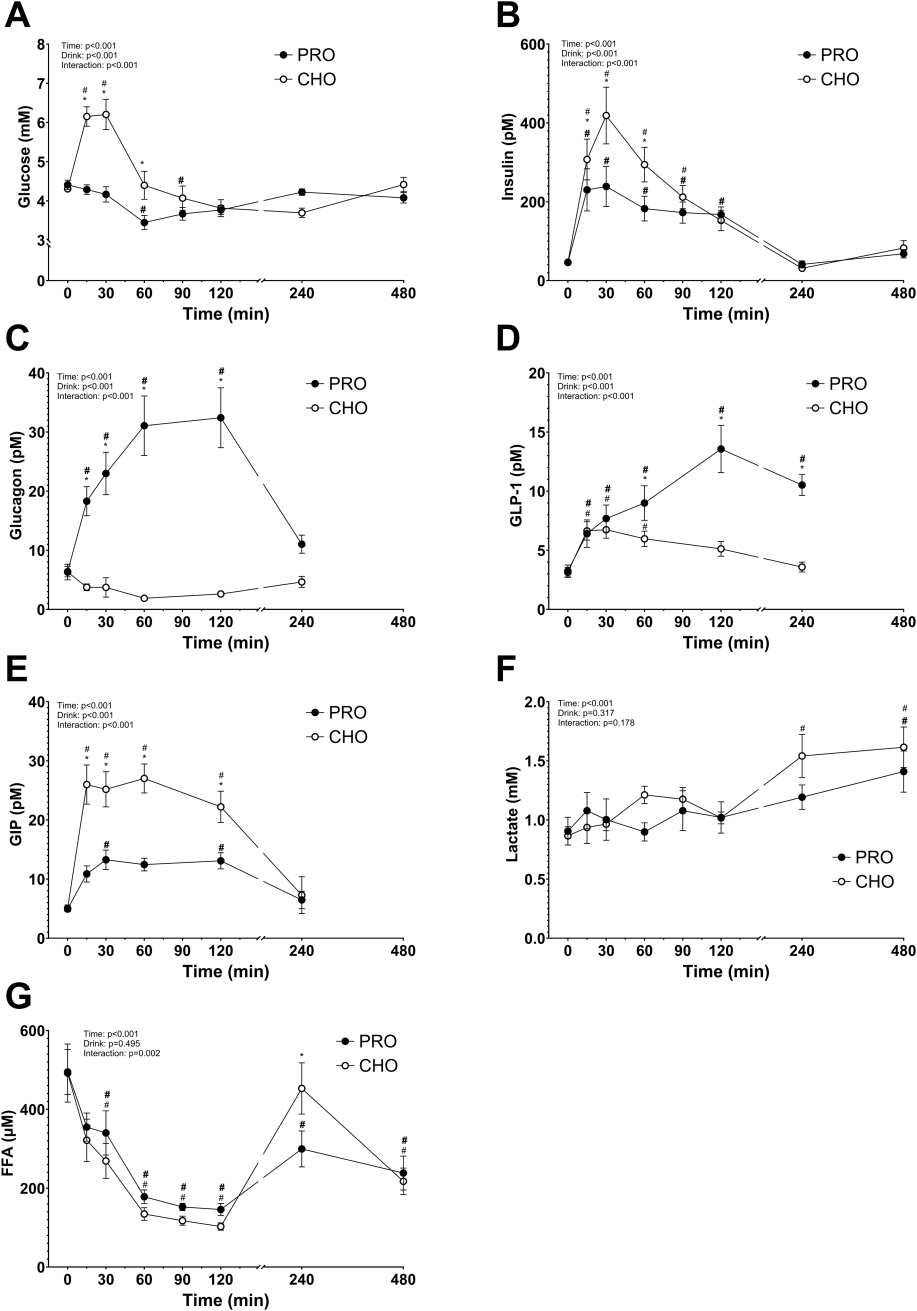
Plasma metabolites and hormones after isocaloric intake of PRO and CHO. **(A)** Plasma glucose concentration (mM). **(B)** Plasma insulin concentration (pM). **(C)** Plasma glucagon concentration (pM). **(D)** Plasma GLP-1 concentration (pM). **(E)** Plasma GIP concentration (pM). **(F)** Plasma lactate concentration (mM). **(G)** Plasma free fatty acids concentration (μM). Values are means ± SEM. * p < 0.05, time points with differences between carbohydrate (CHO) and protein (PRO). # p < 0.05, differences with t= 0 min in CHO. # p < 0.05, differences with t= 0 min in PRO.

After ingesting the drink, plasma glucose concentration increased in CHO while it decreased in PRO (time effect, p<0.001; effect size PRO *d* = 0.40 small, CHO *d* = 1.44 very large) (**Figure 2A**). This led to a significantly higher plasma glucose concentration in CHO at 15 min (p<0.001), 30 min (p<0.001), and 60 min (p<0.001) following ingestion of the drink compared to in PRO. The plasma glucose concentration in CHO then decreased and was no longer significantly different than in PRO.

Area under the curve (AUC) for glucose concentrations in the two hours following the ingestion of the drinks was significantly higher in CHO than in PRO (CHO, 575 ± 32 arbitrary units; PRO, 461 ± 16 arbitrary units; p<0.001).

### 3.2 Plasma insulin concentration

Following consumption of the drink, plasma insulin concentration increased in both CHO and PRO (time effect, p<0.001) but more in CHO (drink effect, p<0.001; effect size PRO *d* = 1.49 very large, CHO *d* = 1.97 very large) (**Figure 2B**). Resulting in a significantly higher concentration of insulin at 15 min (p=0.047), 30 min (p<0.001), and 60 min (p<0.001) after CHO compared to PRO ingestion. AUC for insulin concentrations in the two hours following the ingestion of the drinks was significantly higher in CHO than in PRO (p<0.001).

### 3.3 Plasma glucagon concentration

After ingesting the drink, plasma glucagon concentration increased in PRO (drink effect, p<0.001; effect size PRO *d* = 1.64 very large, CHO *d* = 0.95 very large) and decreased in CHO (**Figure 2C**). Resulting in a significantly higher concentration of glucagon at 15 min (p=0.001), 30 min (p<0.001), 60 min (p<0.001), and 120 min (p<0.001) after PRO compared to CHO ingestion.

### 3.4 Plasma GLP-1 concentration

Post-drink ingestion, plasma GLP-1 concentration increased both in PRO and CHO (time effect, p<0.001; effect size PRO *d* = 1.83 very large, CHO *d* = 0.76 large) (**Figure 2D**). At 120 min after ingesting the drink, plasma GLP-1 concentration was no longer significantly different from baseline in CHO. However, the plasma GLP-1 concentration continued to increase in PRO and was significantly elevated 240 min after ingesting the drink. Leading to a significantly higher concentration of GLP-1 at 60 min (p=0.017), 120 min (p<0.001), and 240 min (p<0.001) after PRO compared to CHO ingestion.

### 3.5 Plasma GIP concentration

After ingestion of the drink, plasma GIP concentration increased both in PRO and CHO (time effect, p<0.001; effect size PRO *d* = 1.32 very large, CHO *d* = 1.67 very large) (**Figure 2E**). The increase was however much higher in CHO resulting in a significantly higher concentration at 15 min (p<0.001), 30 min (p<0.001), 60 min (p<0.001), and 120 min (p=0.003) after CHO ingestion compared to PRO ingestion.

### 3.6 Plasma lactate concentration

After the drink ingestion, the plasma lactate concentration remained constant both in CHO and PRO, with no difference between the two conditions (drink effect, p=0.317) (**Figure 2F**).

### 3.7 Plasma free fatty acid concentration

Plasma FFA concentration decreased significantly in both PRO and CHO during the first 120 min (time effect, p<0.001; effect size PRO *d* = 1.92 very large, CHO *d* = 1.82 very large), followed by a subsequent increase observed up to 240 min post-drink ingestion (**Figure 2G**). There was no significant difference between the two conditions (p=0.495), but FFA concentration was significantly higher at 240 min after CHO ingestion compared to PRO ingestion (p=0.002).

### 3.8 Plasma AA concentration

**Figure 3:**
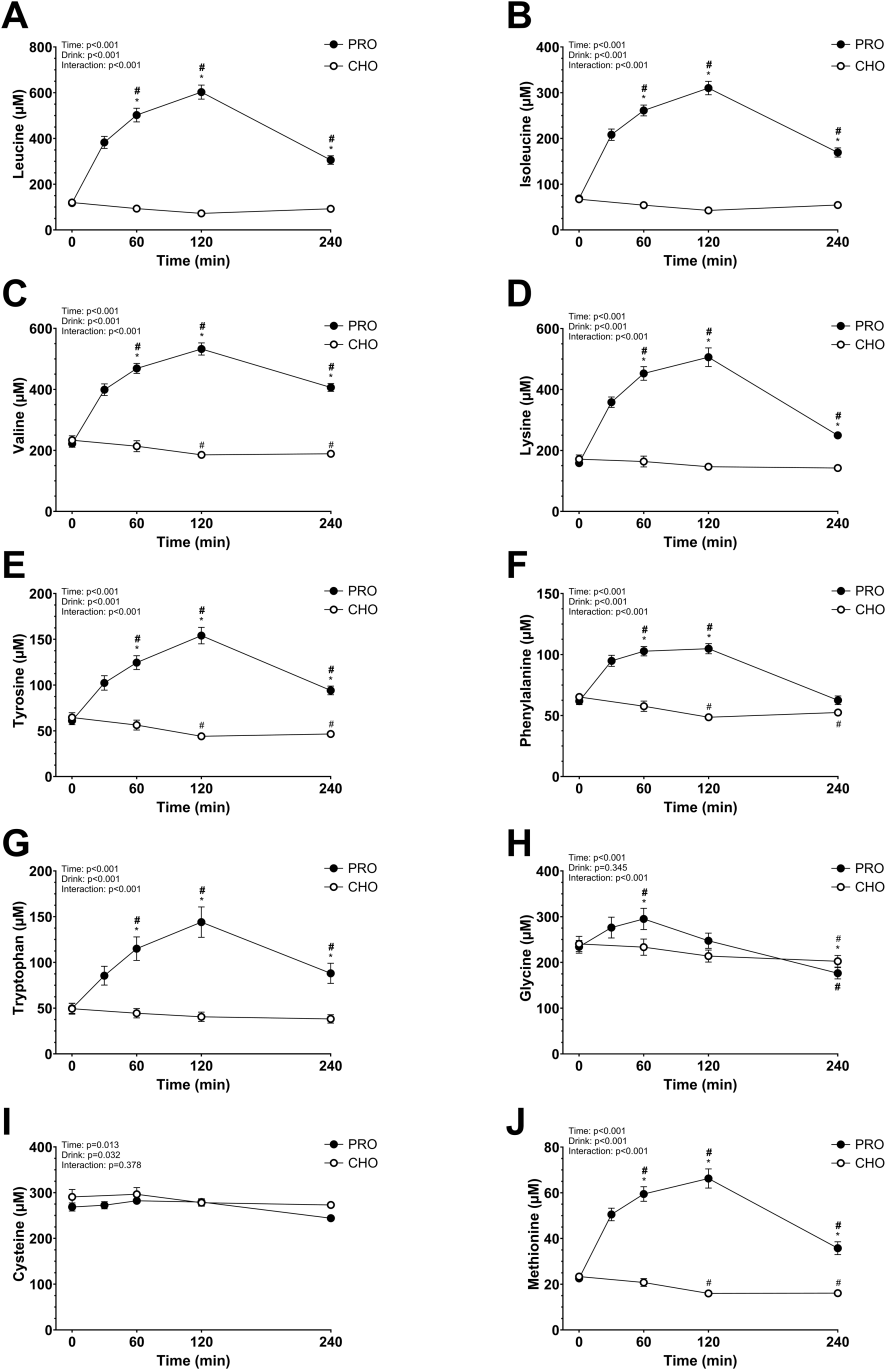
Plasma amino acids after isocaloric intake of PRO and CHO. **(A)** Plasma leucine concentration (µM). **(B)** Plasma isoleucine concentration (µM). **(C)** Plasma valine concentration (µM). **(D)** Plasma lysine concentration (µM). **(E)** Plasma tyrosine concentration (µM). **(F)** Plasma phenylalanine concentration (µM). **(G)** Plasma tryptophan concentration (µM). **(H)** Plasma glycine concentration (µM). **(I)** Plasma cysteine concentration (µM). **(J)** Plasma methionine concentration (µM). Values are means ± SEM. * p < 0.05, time points with differences between carbohydrate (CHO) and protein (PRO). # p < 0.05, differences with t= 0 min in CHO. # p < 0.05, differences with t= 0 min in PRO.

**Figure 4:**
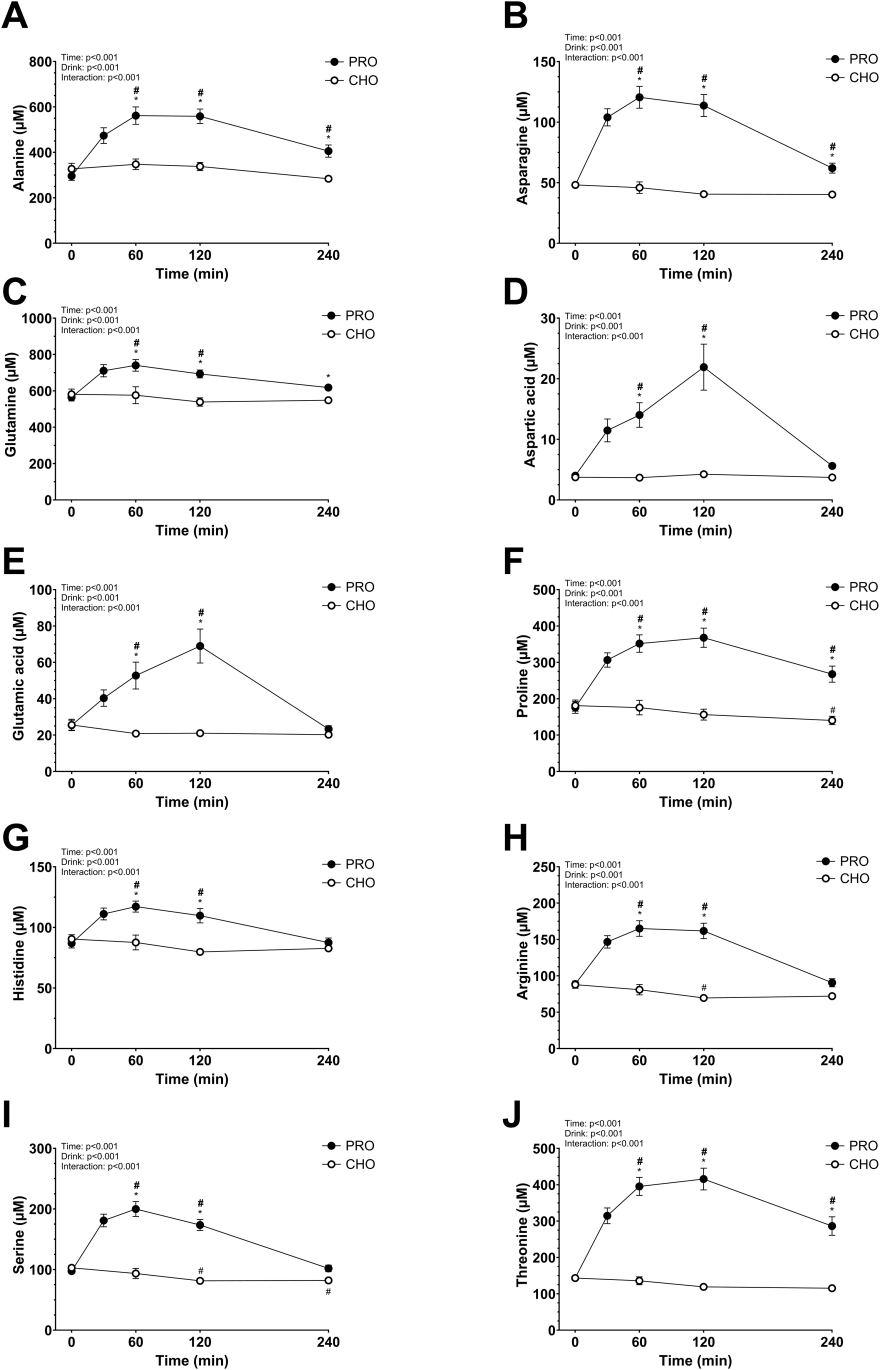
Plasma amino acids after isocaloric intake of PRO and CHO. **(A)** Plasma alanine concentration (µM). **(B)** Plasma asparagine concentration (µM). **(C)** Plasma glutamine concentration (µM). **(D)** Plasma aspartic acid concentration (µM). **(E)** Plasma glutamic acid concentration (µM). **(F)** Plasma proline concentration (µM). **(G)** Plasma histidine concentration (µM). **(H)** Plasma arginine concentration (µM). **(I)** Plasma serine concentration (µM). **(J)** Plasma threonine concentration (µM). Values are means ± SEM. * p < 0.05, time points with differences between carbohydrate (CHO) and protein (PRO). # p < 0.05, differences with t= 0 min in CHO. # p < 0.05, differences with t= 0 min in PRO.

The plasma AA concentrations increased significantly in PRO compared to in CHO for most of the AA measured (drink effect, p<0.001; effect size *d* = 2.15 very large). All data are shown in **Figures 3** and **4**. Some AA concentrations were significantly higher in PRO than in CHO at 60 min, 120 min, and 4 h after ingesting the drink. While others only had significantly higher concentrations in PRO compared to in CHO at 60 min and 120 min after ingesting the drink.

### 3.9 Urinary nitrogen excretion and nitrogen balance

**Figure 5:**
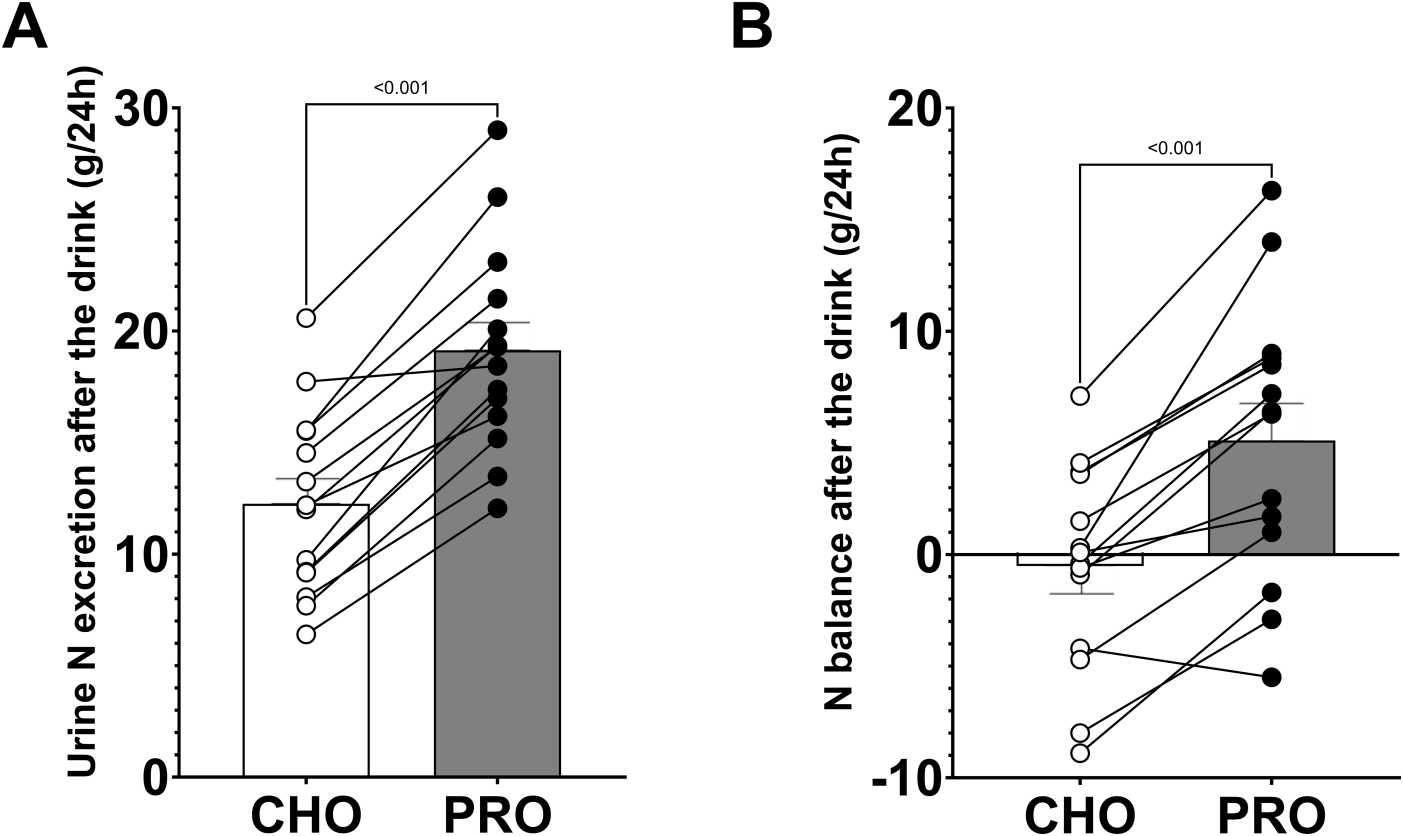
Urinary nitrogen excretion and nitrogen balance during the 24 hours following the ingestion of isocaloric intake of PRO and CHO. **(A)** Urinary nitrogen excretion after the ingestion of the drinks. **(B)** Nitrogen balance after the ingestion of the drinks. Values are means ± SEM.

During the 24 hours following the ingestion of the drinks, urinary nitrogen excretion was significantly higher in PRO compared to in CHO (p<0.001; PRO, 19.14 ± 1.24 g; CHO, 12.26 ± 1.11 g) (**Figure 5A**). Additionally, urinary nitrogen excretion was significantly higher in PRO after normalizing by bodyweight (p<0.001; PRO, 255.9 ± 14.0 mg·kg^-1^; CHO, 163.0 ± 12.9 mg·kg^-1^).

Nitrogen intake was calculated to be 29.6 ± 2.2 g (2.44 ± 0.12 g·kg^-1^ protein) in PRO and 15.2 ± 1.8 g (1.24 ± 0.12 g·kg^-1^ protein) in CHO. The nitrogen balance after the ingestion of the test drinks was positive in PRO (5.1 ± 1.7 g) and neutral in CHO (-0.5 ± 1.2 g), with a significant difference between the two conditions (p<0.001) (**Figure 5B**).

**Figure 6:**
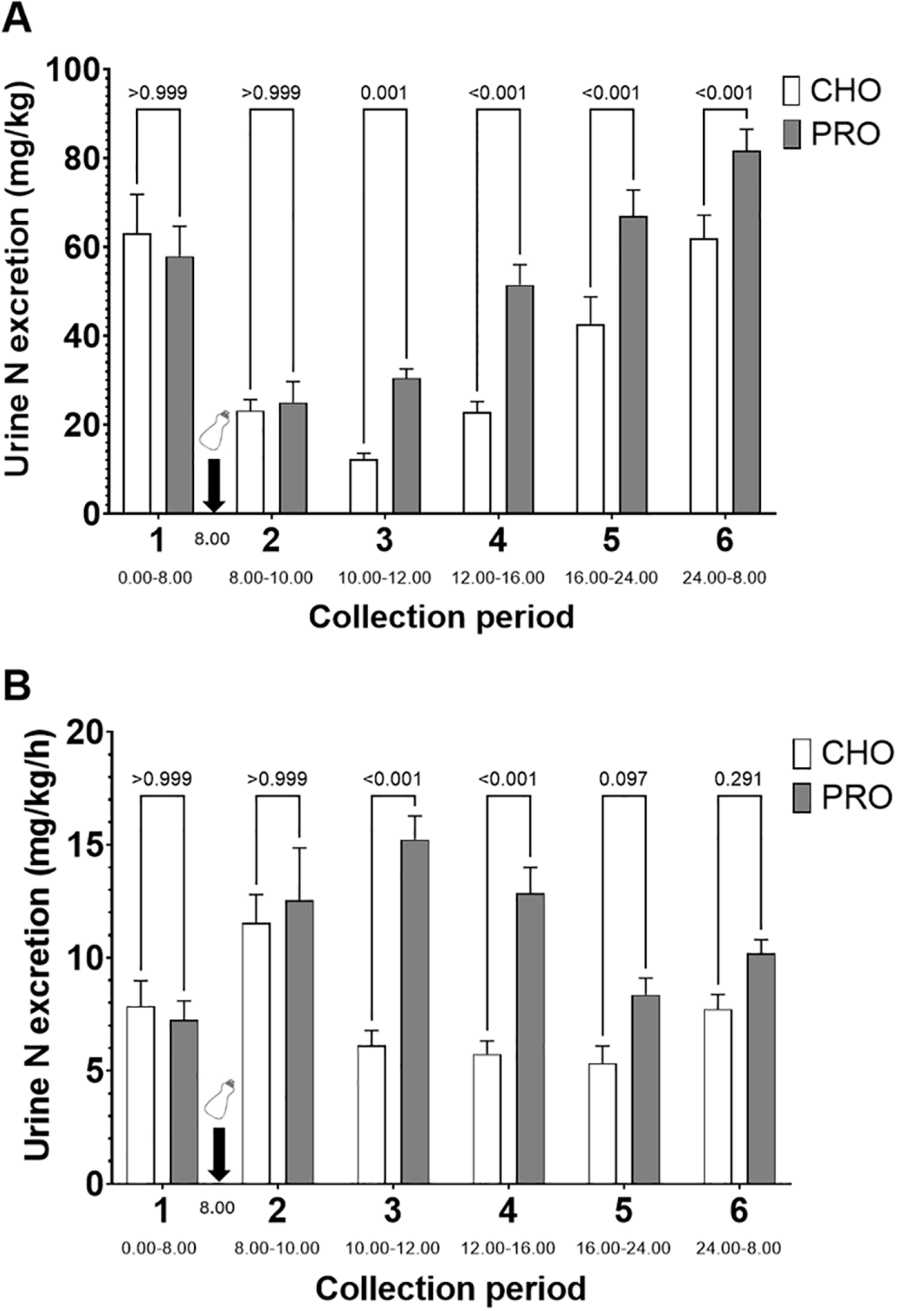
Time course of urinary nitrogen excretion after isocaloric intake of PRO and CHO. **(A)** Quantity of urinary nitrogen excreted per period normalized by body weight. **(B)** Quantity of urinary nitrogen excreted per period normalized by body weight and by time. The time of day for the urine collection period is indicated below the name of the collection period. Values are means ± SEM.

Urinary nitrogen excretion normalized by bodyweight was significantly higher in PRO than CHO during periods 3 (p=0.001; effect size *d* = 0.65 large), 4 (p<0.001; effect size *d* = 1.03 very large), 5 (p<0.001; effect size *d* = 0.87 very large), and 6 (p<0.001; effect size *d* = 0.71 large) (**Figure 6A**). Additionally, when normalized by time, urinary nitrogen excretion was significantly higher in PRO than CHO during periods 3 (p<0.001; PRO, 15.2 ± 1.0 mg kg^-1^ h^-1^; CHO, 6.1 ± 0.7 mg kg^-1^ h^-1^; effect size *d* = 1.84 very large) and 4 (p<0.001; PRO, 12.9 ± 1.1 mg kg^-1^ h^-1^; CHO, 5.7 ± 0.6 mg kg^-1^ h^-1^; effect size *d* = 1.44 very large) (**Figure 6B**). It tended to be significantly higher in PRO than CHO during period 5 (p=0.097; PRO, 8.4 ± 0.7 mg kg^-1^ h^-1^; CHO, 5.3 ± 0.8 mg kg^-1^ h^-1^).

### 3.10 Percentage of nitrogen excreted from the PRO drink

**Figure 7:**
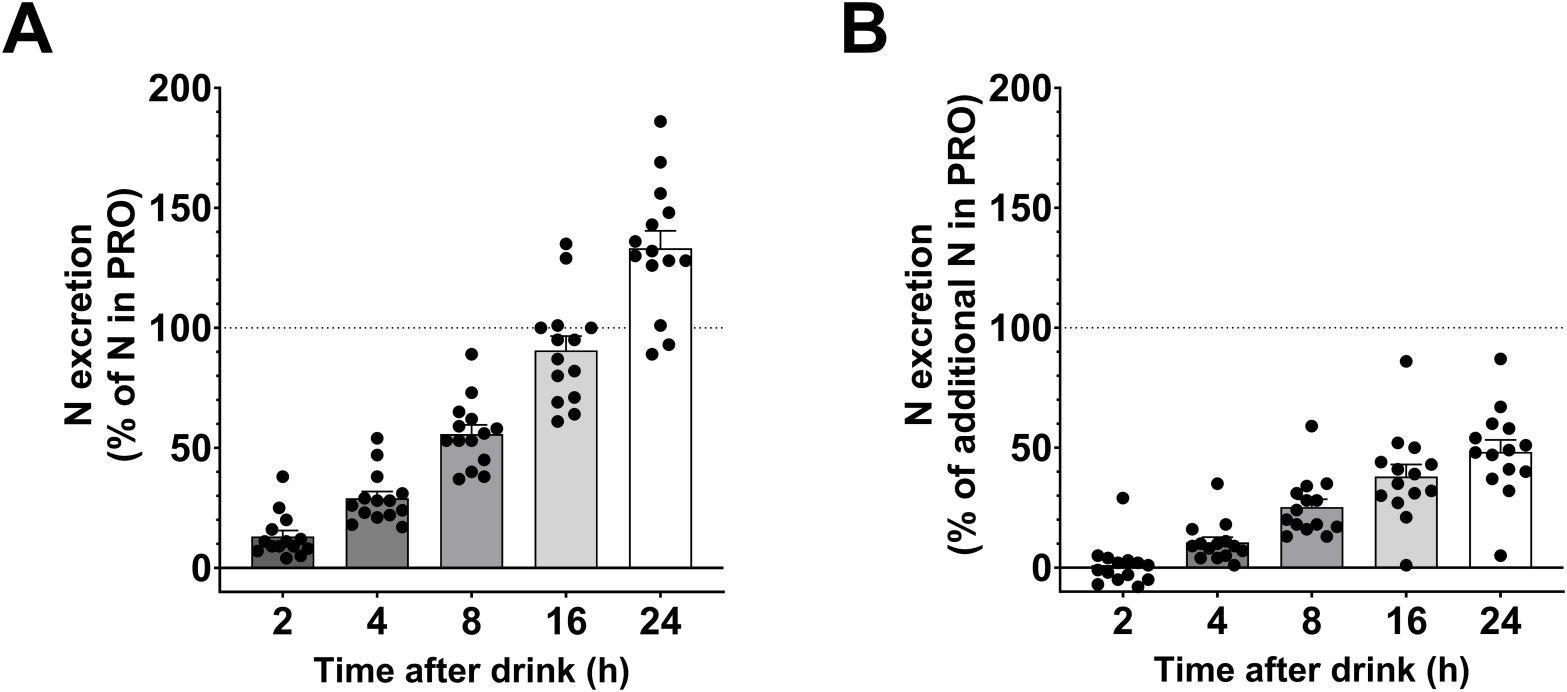
Relative nitrogen excretion after the PRO drink. **(A)** Percentage of urinary nitrogen excreted relative to the amount of nitrogen in the PRO drink (urinary nitrogen excretion in PRO divided by amount of nitrogen in the PRO drink). **(B)** Percentage of urinary nitrogen excreted above urinary nitrogen excretion following CHO intake (urinary nitrogen excretion in PRO minus urinary nitrogen excretion in CHO, all divided by amount of nitrogen in the PRO drink). Values are means ± SEM.

When considering the absolute urinary nitrogen excretion, 56 ± 4% of the nitrogen ingested in the PRO drink was excreted after 8 hours and 133 ± 7% after 24 hours (**Figure 7A**). When subtracting urinary nitrogen excretion in CHO, these figures decreased to 25 ± 3% after 8 hours and 48 ± 5% after 24 hours (**Figure 7B**).

### 3.11 Correlation between the protein intake the day before and the urinary nitrogen excretion during the testing period

**Figure 8:**
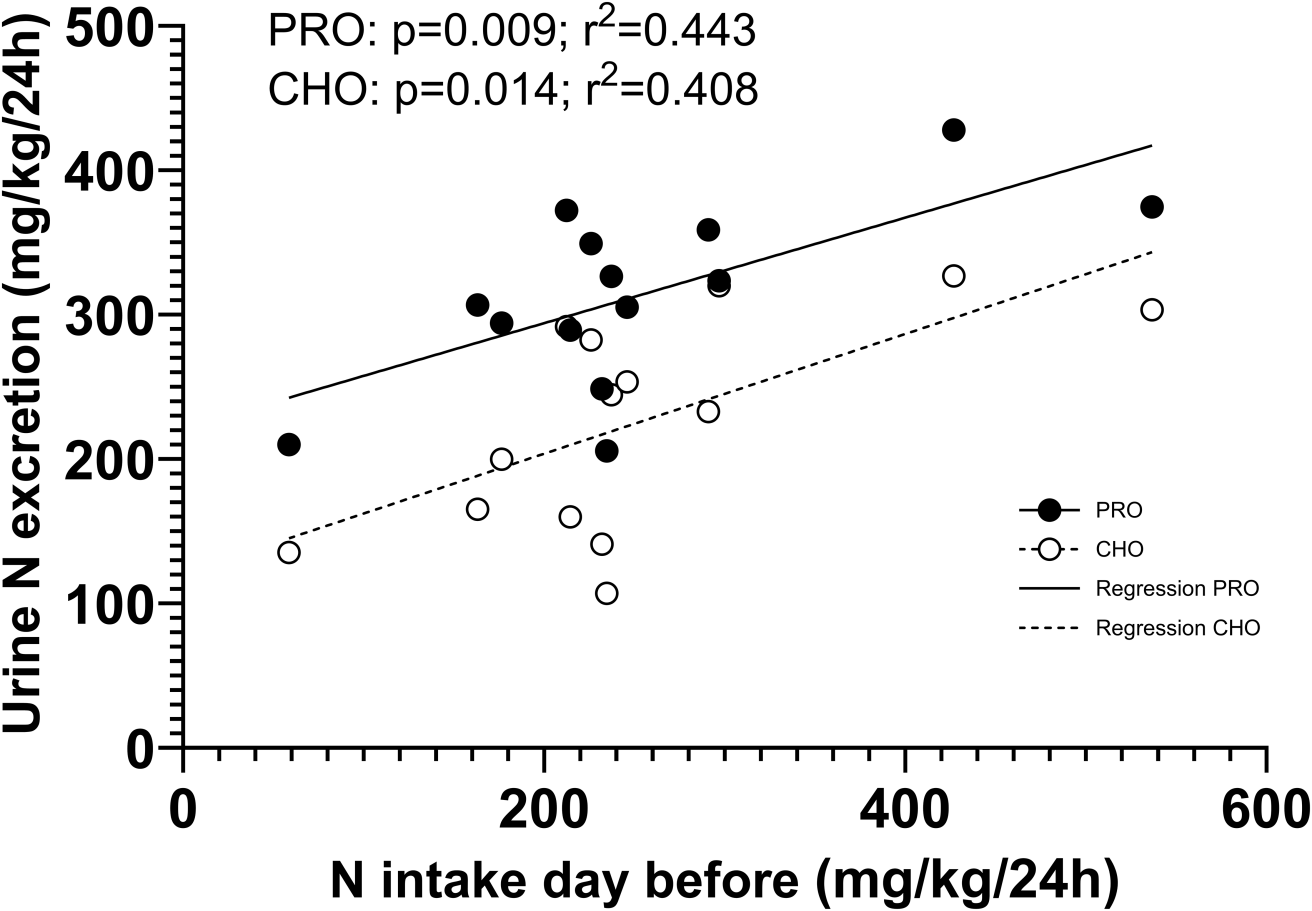
Correlation between the protein intake the day before and the urinary nitrogen excretion during all six periods.

A significant linear correlation was observed between the urinary nitrogen excretion and the nitrogen intake on the previous day, during all urine collection periods (**Figure 8**).

## 4 Discussion

This study investigated the plasma hormone and metabolite responses as well as urinary nitrogen excretion following the isocaloric intake of protein-only (PRO) versus carbohydrate-only (CHO), which served as a control. We found a significant insulin response in PRO despite a decrease in plasma glucose levels. Interestingly, plasma GLP-1 increased twice as much after PRO intake compared to CHO, whereas the opposite was observed for GIP. Furthermore, PRO ingestion increased urinary nitrogen excretion, mainly from 2 to 8 hours post-intake of the drink, but still elevated up to 24 hours after.

### 4.1 Plasma responses of metabolites and hormones

Numerous studies have examined the plasma responses of metabolites and hormones after intake of carbohydrate-only drinks (11, 12, 16, 19, 43). In agreement with previous studies, we report that plasma glucose and insulin levels increased rapidly after CHO intake, peaking at 30 minutes before decreasing. Plasma glucose returned to basal levels after 60 minutes, while insulin remained elevated for 120 minutes and returned to fasting level after 240 minutes. As expected, plasma glucagon and FFA concentrations decreased during the first 120 minutes after CHO intake and returned to basal level after 240 minutes (16, 19). Additionally, the incretins GLP-1 and GIP concentrations followed the expected patterns, peaking at 30–60 minutes after CHO ingestion and returning to pre-ingestion levels by 240 minutes (12, 16, 19). Most plasma AAs remained unchanged but some decreased after CHO intake.

**Figure 9:**
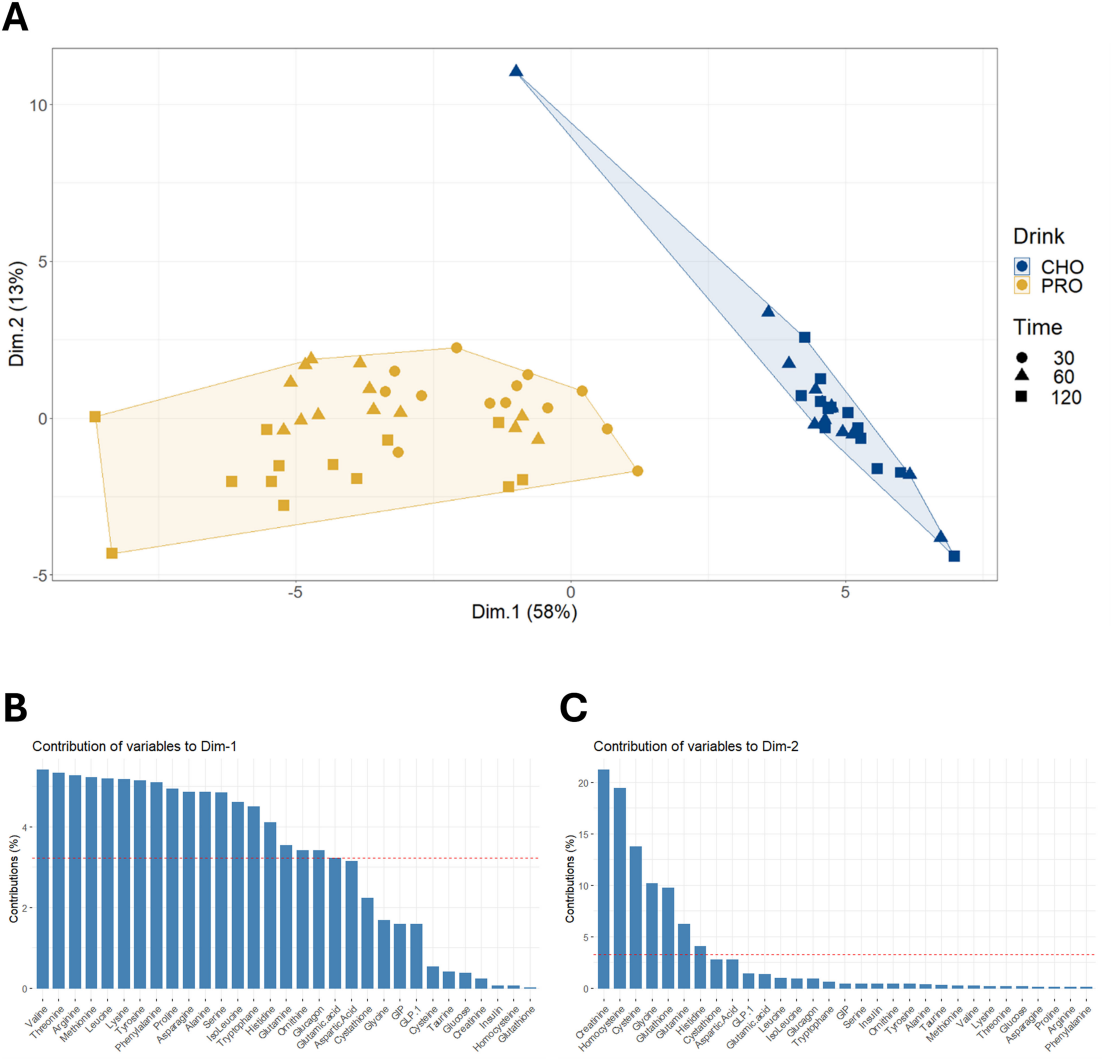
Principal component analysis on changes in plasma concentrations of amino acids and hormones. **(A)** Hierarchical clustering on principal component analysis performed on changes in plasma concentrations of amino acids and hormones from pre-ingestion to 30, 60, and 120 minutes post-drink ingestion in both PRO and CHO. **(B)** Description of the contribution of variables to dimension 1. **(C)** Description of the contribution of variables to dimension 2.

Surprisingly, only a few studies have investigated hormonal and metabolite responses after intake of protein alone. The primary objective of the current study was to explore the plasma responses of hormones, AAs, and incretin hormones following the intake of a large dose of protein-only. In PRO, the insulin response peaked at about 50% of the CHO response after 15–30 minutes, then gradually decreased. The significant insulin response could not be attributed to plasma glucose, as plasma glucose concentration remained at basal levels for the first 30 minutes after PRO ingestion and was significantly decreased at both 60 and 90 minutes post-ingestion. Concurrently, almost all plasma AAs increased and reached their highest level after 60 or 120 minutes. AA-induced stimulation of insulin secretion has been reported both *in vitro* (25) and *in vivo* (12, 44, 45). The principal component analysis of plasma metabolites at the time points when AAs were measured showed clearly clusters (**Figure 9**), with CHO distinctly separated from PRO. The first dimension, accounting for 58% of the total variation, was primarily composed of AAs, each contributing similarly to this dimension. In PRO, the increase in insulin concentration correlated with the increase in arginine, asparagine, cysteine, glutamine, and phenylalanine concentrations (p<0.05). All these AAs have been reported to stimulate insulin secretion (12, 44, 45) through different mechanisms (25). Therefore, the data in the present study indicate that many AAs contributed to the insulin response.

Changes in insulin concentration in PRO were also significantly correlated with the increase in GLP-1 (p=0.033), but not the increase in GIP (p=0.855). Our results suggest that GLP-1 is the sole incretin investigated that is likely to amplify the insulin response to AAs. Previous studies support these findings, showing that the infusion of glucose and GLP-1 significantly increased plasma insulin levels compared to glucose infusion alone, whereas infusing glucose and GIP at physiological levels was less effective (46). The effect of GLP-1 in combination with AA infusion has not been studied. Notably, 240 minutes after PRO intake, GLP-1 and several AAs remained elevated, while plasma insulin had returned to fasting level.

Plasma GLP-1 responses were similar in PRO and CHO the first 30 minutes post-ingestion. However, after 30 minutes GLP-1 continued to rise in PRO and was 3-fold elevated after 240 minutes compared to when CHO was ingested. We are the first to report such a prolonged elevation of plasma GLP-1, but our observation is to some degree supported by previous studies where intake of protein led to an elevation of GLP-1 (16, 29, 30). In contrast to GLP-1, plasma GIP increased 2-fold more in CHO compared to in PRO and both returned to basal levels after 240 min, which is in agreement with prior research (16). The differences in GLP-1 and GIP responses to the drinks are enigmatic, but could be attributed to their distinct sites of secretion and underlying mechanisms. GIP is secreted by K cells in the duodenum and jejunum after food intake, while GLP-1 is secreted by L cells in the ileum and colon (47–49). *In vitro* studies have demonstrated that several AAs including glutamine, arginine, proline, tryptophan and phenylalanine, effectively stimulate the release of both GLP-1 (50, 51) and to some extent GIP (52). The whey protein in PRO contains all required AAs for GLP-1 release (53, 54), but must undergo digestion before absorption. The later GLP-1 peak after PRO intake has been reported previously (16, 30) and may result from slower digestion of protein compare to carbohydrates. The physiological role of high plasma GLP-1 long after PRO intake remains unclear and warrants further investigation. The physiological roles of GLP-1 have attracted large interest and the incretin contributes to insulin secretion during glucose intake, and gastric motility, satiety and hepatic metabolism (55). The effectiveness of GLP-1 agonists to reduce body weight have attracted much attention and they are now used to treat obesity (56–58). Given GLP-1’s crucial role in regulating food intake and satiety (59, 60), it would have been of interest to evaluate satiety before the lunch. However, we were surprised by the prolonged elevation of GLP-1 in PRO, and, unfortunately, did not consider evaluating satiety.

Generally, plasma AA concentrations increase after the ingestion of a protein drink and typically return to basal levels within 2–3 hours (23, 61, 62). However, in the present study, only half of these concentrations had returned to resting levels by the 4-hour mark. The extended time before returning to basal levels in our study may be due to the higher protein quantity consumed. Notably, levels of alanine, asparagine, isoleucine, leucine, lysine, methionine, proline, threonine, tryptophan, tyrosine, and valine remained elevated beyond this 4-hour period. Interestingly, cysteine did not increase as other AAs, despite its content in the ingested whey protein (2.4 g per 100 g of whey protein powder). As expected, the branched-chain AAs leucine, isoleucine, and valine showed a considerable increase in plasma concentration at peak levels. This aligns with the fact that, unlike most AAs, branched-chain AA catabolism initially occurs in the skeletal muscle rather than the liver, due to low hepatic activity of the degradation enzyme branched-chain AA aminotransferase (63). Glutamine and alanine had a moderate increase in plasma concentration which may be explained by their function as major substrates utilized by intestinal cells, leading to a lower quantity reaching the bloodstream (64, 65).

In the current study, the ingestion of PRO triggered a glucagon response, which is known to inhibit glycolysis and stimulate the liver to produce glucose from glycogen and non-carbohydrate sources through gluconeogenesis (31). Similarly, it has been demonstrated that ingestion of a whey protein drink stimulates hepatic gluconeogenesis (66).

### 4.2 Urinary nitrogen excretion and the time resolution

A major goal of the present study was to investigate the time-course of nitrogen excretion following the consumption of a large amount of protein only. Given the limited capacity to store AAs in the human body (32, 33), excess AAs are metabolized in the liver and the nitrogen from AAs is incorporated into urea before being excreted mainly in urine. In our study design, CHO can be used as a reference for nitrogen excretion in order to account for obligatory nitrogen loss and to minimize the influence of factors such as energy intake and prior protein intake on urinary nitrogen excretion.

Although urinary nitrogen excretion did not significantly increase during the first 2 hours after PRO ingestion compared to the CHO control, it was higher during this period relative to the morning control period prior to PRO consumption. Our study design does not clarify whether this was due to delayed nitrogen excretion or increased protein degradation in CHO during this period, making the difference less noticeable. The rise in urinary nitrogen excretion in CHO could be attributed to diurnal variations (67–70) or the ingestion of a fluid by fasting participants (71).

During the first 8 hours, nitrogen excretion in PRO corresponded to 56% of the ingested protein in the PRO drink. After subtracting urinary nitrogen excretion in CHO, we found that 25% of the nitrogen ingested in PRO was excreted in urine after 8 hours, and 48% after 24 hours. This indicates that approximately half of the ingested protein remains not excreted after one day. We have investigated nitrogen excretion in several studies (24, 38, 72–74) but we have been uncertain about the time resolution of nitrogen excretion. The data in the present study clearly shows that a high intake of whey protein does not result in an increase in nitrogen excretion during the first 2 hours. However, after 2 hours there was a doubling in urinary nitrogen excretion, followed by a less prolonged increase in the following hours. Moreover, nitrogen excretion during all periods after intake of both PRO and CHO correlated with the reported protein intake from the previous day, highlighting a delay in nitrogen excretion. These findings align with previous studies investigating nitrogen balance with high and low protein intake that showed that protein metabolism is influenced over periods of days, as is evident by the 3-day habituation period required to achieve nitrogen balance when changing protein intake (75, 76). These findings highlight that it is difficult to study nitrogen excretion in studies where protein intakes vary.

### 4.3 Strengths and limitations

The strengths of this study include its double-blinded, randomized, balanced, cross-over design and the standardization of the diet the day before and during the protocol. Allowing participants to consume their habitual protein intake minimized the likelihood that observed differences were due to changes in protein consumption (75). Additionally, including both genders enabled the study of associations across a broader range of variations, such as lean body mass.

Interpreting plasma AAs is challenging. A limitation of our study is that we did not measure AA absorption or metabolic fluxes. The surprising finding that plasma AAs remained elevated 4 hours after PRO intake, makes this limitation critical in interpreting the data. It will be important to investigate whether absorption or AA disposal are responsible for prolonged elevation of plasma AAs. Even at peak plasma concentration, the increase in plasma AAs represented only a small fraction (0.1 and 4.0%) of the total amount of AAs ingested, highlighting that tissue-specific uptake and metabolism of AAs will be important to characterize. We cannot determine if the low peak plasma concentrations were due to slow absorption from the intestine or rapid removal by tissues such as the liver or skeletal muscles. Ingesting a large dose of AAs in a short time may have saturated the body’s capacity to process them. However, we cannot test this hypothesis without measuring AA fluxes. Another limitation is that we did not measure nitrogen excretion in feces or sweat. Previous research shows that increased protein intake either has no effect on fecal nitrogen excretion (3, 77) or results in a small increase in fecal nitrogen excretion (4, 78). Whey protein is normally considered to be absorbed well (79), and we have no indications that this is not the case in the present study, as presented in our data on plasma AA concentrations. Sweat nitrogen excretion is also minimal in resting condition (4, 80). Thus, while we cannot fully exclude minor contributions of fecal and sweat nitrogen excretion, it is unlikely to significantly impact the overall interpretation of our findings.

The findings of the present study cannot be directly translated to other populations, such as older adults, sedentary individuals, those with chronic illnesses (e.g., diabetes), or people with lower fitness levels. These groups exhibit unique metabolic and hormonal responses to nutrient intake that may influence their responses to interventions. Future research should aim to include a broader range of participants to better understand the effectiveness of our findings across various demographics. Regarding the increased hormone secretion after PRO ingestion, we cannot entirely rule out the influence of low levels of nonprotein components in PRO (fat 0.4% (0.3 g) and carbohydrate 2.4% (1.8 g)), but this is unlikely to explain the hormonal responses observed since FFA responded similarly and plasma glucose decreased during PRO intake.

### 4.4 Perspectives

From a nutrition and health perspective, our findings underscore the contrasting effects of protein and carbohydrates on incretin hormone secretion. The pronounced increase in GLP-1 secretion with PRO intake suggests that incorporating higher protein foods into meals may enhance satiety and improve glycemic control, potentially benefiting individuals with insulin sensitivity issues or those aiming for weight management (81–83). Furthermore, the insulin response associated with PRO intake, independent of glucose levels, highlights the need for a balanced dietary approach that prioritizes protein, particularly for those looking to optimize metabolic health. Given the prolonged increased urinary nitrogen excretion observed following protein ingestion, we recommend that dietary guidelines consider protein intakes over several days in order to consider the extended time resolution of the protein metabolism.

## 5 Conclusion

This study demonstrated that isocaloric high intake of either protein-only or carbohydrate-only significantly increased incretin levels, but with differing effects. Protein intake led to a more pronounced increase in GLP-1 secretion, whereas carbohydrate intake resulted in significant GIP secretion. Both conditions, however, induced an insulin response. Notably, the insulin response to protein intake was independent of glucose levels, suggesting that dietary amino acids may stimulate insulin release through an amplified GLP-1 response. The extent to which this is the sole mechanism requires further investigation. Additionally, our findings showed that protein ingestion alone significantly elevated urinary nitrogen excretion, primarily between 2 to 8 hours after intake, with elevated levels persisting up to 24 hours later.

## Data Availability

The original contributions presented in the study are included in the article/**Supplementary Material**. Further inquiries can be directed to the corresponding author.
